# Components Affecting the Promotion of COVID-19 Disease Prevention Behaviors among Iranian Students: A Qualitative Study by Using the PRECEDE Model

**DOI:** 10.1155/2022/7807401

**Published:** 2022-08-05

**Authors:** Mehdi Layeghiasl, Mohammad Hossein Kaveh, Masoud Karimi, Alireza Mirahmadizadeh

**Affiliations:** ^1^Health Education and Health Promotion, Student Research Committee, Department of Health Promotion, School of Health, Shiraz University of Medical Sciences, Shiraz, Iran; ^2^Research Center for Health Sciences, Institute of Health, School of Health, Shiraz University of Medical Sciences, Shiraz, Iran; ^3^Health Education and Promotion, School of Health, Shiraz University of Medical Sciences, Shiraz, Iran; ^4^Non-Communicable Diseases Research Center, Shiraz University of Medical Sciences, Shiraz, Iran

## Abstract

**Background:**

COVID-19 is a social health problem. Several risk factors threaten students, and schools can provide a suitable environment for managing and performing health promotion programs. Given the extensive spread of the disease and the existence of multilevel components affecting the adoption of preventive behaviors, understanding the views and opinions of the audience about the barriers and facilitators affecting the behavior using qualitative studies can be one of the ways to have a successful intervention.

**Materials and Methods:**

This was a qualitative study performed by a directed content analysis method while using the PRECEDE model (predisposing, reinforcing, enabling constructs in educational diagnosis and evaluation). In total, 38 individuals (teachers, parents, and students) were entered into the study using the purposive sampling method. Data were collected by semistructured interviews, and the implemented data were systematically classified into five stages (condensed meaning units, code, subcategory, category, and theme) and were arranged and analyzed.

**Results:**

The findings are classified into three themes of predisposing, enabling, and reinforcing factors. In addition, perceived sensitivity, perceived intensity, mental beliefs, behavior outcome evaluation, and perceived power were considered as subcategories of predisposing factors, whereas normative beliefs and motivation for adherence to protocols were subcategories of reinforcing factors, and control beliefs were subcategories of enabling factors.

**Conclusion:**

Our findings could be used as a guide to design educational interventions aiming at promoting COVID-19 prevention behaviors in schools.

## 1. Introduction

There are various types of communicable acute respiratory diseases caused by the coronavirus, which have made extreme concerns worldwide due to their extensive prevalence [1]. The first case of a respiratory disease (COVID-19) caused by a new type of coronavirus (SARS-CoV-2) was detected in Wuhan, China, in late December 2019. The World Health Organization (WHO) declared the outbreak a Public Health Emergency of International Concern on 30 January 2020 [2]. This epidemic has had numerous social, economic, political, and educational outcomes in the world, and governments have been forced to adopt different policies and strategies, such as school closures [3]. Because of schools, hospitals, workplaces, prisons, universities, and markets, the diverse environments are emphasized by WHO in health promotion programs [4]. Meanwhile, schools have been regarded as one of the basic and effective environments in health promotion programs [5]. The Center for Disease Control and Prevention (CDC) has also mentioned that investing in the health of the youth and adolescents in educational environments is one of the most important interventions of health systems [6, 7]. This shows that the cooperation and encouragement of the youth to perform preventive behaviors can inhibit the spread of the virus [8]. On the other hand, this allows the establishment of people's health in their everyday living environment, where they learn, work, and play [9].

Therefore, given that students with a population of more than 12 million people make up a significant part of the population of Iran [10], they are affected by multiple risk factors in schools [11]. Overall, schools are one of the appropriate environments for the organization and execution of health promotion programs [12]. On the other hand, participation can be one of the basic components involved in the successful implementation of health promotion interventions. However, there is low participation of adolescents in these programs due to their age [13]. Identification of barriers to the participation of these individuals and identifying the facilitators and promoters of their cooperation with health promotion programs can be one of the effective solutions to attract the participation of this target group and successfully perform interventional programs in this regard [14]. As a result, recognizing the audiences of the interventions individually, collectively, and organizationally and determining the factors related to health behaviors can lead to the cooperation of these people with health promotion programs [15, 16]. In fact, finding reasons to focus on the issue and identifying obstacles and solutions for them are effective strategies in this respect [16, 17]. Understanding these key factors is possible by doing qualitative research as a necessary step [18]. In general, qualitative research is used to have a deep understanding of the target population's viewpoints and opinions in the texture and environment [19]. Qualitative content analysis is one of the methods of qualitative research, which is also used in general health studies and provides a new insight with a practical guide to action [20]. Qualitative content analysis has several approaches to qualitative data analysis, including the directed content analysis, which is used to present more descriptions of the existing theories [20, 21]. Therefore, there is a need to use an appropriate model to carry out the systematic process of needs assessment. In this regard, the PRECEDE model is a population-based epidemiological planning framework that has various ecological aspects and systematically enables planners to identify health issue features [22]. This model analyzes and describes the health problem and evaluates predisposing, reinforcing, and enabling constructs as factors affecting disease prevention and control [12, 23].

Given the unknown future of the COVID-19 disease, schools and students are, respectively, one of the best environments and target groups for promoting COVID-19 prevention behaviors [24]. Since various factors determine the adoption of COVID-19 prevention behaviors [3] and given the importance of the use of these behaviors sensitive and crowded environments such as schools, the present study is aimed at determining and identifying factors affecting the promotion of COVID-19 prevention behaviors in boys' schools (seventh and eighth grades) with the cooperation of students, as well as their parents and teachers by using the directed content analysis and the PRECEDE model in Yasuj, Iran.

## 2. Materials and Methods

This was a qualitative study performed by a directed content analysis method based on the PRECEDE model. In directed content analysis, existing theories or the results of previous research form the basis of the analysis. Therefore, given that the directed content analysis is used to more explain the existing theories or conceptually expand a theoretical framework, the directed content analysis method was applied based on the PRECEDE model [21].

### 2.1. Participants and Sampling Method

In total, 38 individuals (15 teachers, 10 parents, and 13 students) were enrolled in the study based on the principle of maximum variation of sampling and by using the purposive sampling method [25]. In addition, the participants were selected based on two criteria of experimental fit and good informant, which is known as participant quality [26], in order to provide the best information about the research community. The inclusion criteria were a student, teacher, or parent of a student, age, gender, level of education of parents and teachers, and age and grade of students. Given that the current research laid the foundation for the development of a model for the promotion of COVID-19 prevention behaviors in boys' schools, and since it is difficult to conduct a study by a male researcher in a girls' school due to the cultural conditions of the country, we only focused on male students. Following visiting the healthcare centers of Yasuj and reviewing the medical files of families, the researcher invited key informants who had knowledge of the issue or were affected by it. Notably, informed consent was obtained prior to interviews. Since there is no definite sample size in qualitative research, interviews continued until reaching data saturation, meaning that no new information was achieved after a while [27].

### 2.2. Instrument and Data Collection Method

Data were collected from in-depth interviews, the questions of which were semistructured. The interview guide was compiled with the participation of the research team and using a panel of experts. Because the interviews were semistructured, the questions related to the study objectives and to identify the determinants were designed based on the three constructs of the PRECEDE model, including predisposing, enabling, and reinforcing factors. First, general questions were asked beforehand based on the study objectives and research framework designed. Afterwards, in-depth questions were asked to have a deeper understanding of the subjects' viewpoints. Any questions that would come to the interviewer's mind were also asked during interviews and used in future interviews [28].

Attempts were made to use open questions in order to assess the participants' views on COVID-19 protocols and factors affecting the use of COVID-19 prevention behaviors. In-depth interview guide list was as follows: first, a general question such as “what do you know about COVID-19 disease?” was asked to encourage the participant to talk about the topic. Afterwards, based on the responses received, other questions were asked about the disease complications, transmission methods, prevention techniques, and reasons for the importance of prevention. More details were gradually added to the interviews, such as “could you share your experiences regarding COVID-19 prevention?,” “in your opinion, why do people still avoid the use of COVID-19 prevention protocols, despite all the problems caused by the disease?,” “what are the barriers to the use of COVID-19 prevention behaviors among students?,” “what are the solutions for these problems?,” “what solutions do you suggest to do these behaviors?,” and “do you believe that there are people who can affect students' adherence to protocols? How?” The interviews were conducted individually and with the cooperation of the head of the health center in Yasuj in a separate room. It is notable that the interviewer and interviewee wore masks during the process and observed the appropriate distance, and the room had a proper ventilation system. Each interview lasted 40-60 minutes, and all interviews were recorded following receiving informed consent from the participants.

### 2.3. Data Analysis

Data analysis was carried out manually and by using the qualitative content analysis method. The interviews were recorded verbatim word by word at the end of the process. The texts were read carefully several times to gain a general understanding of them. The sentences that were responses to questions were identified, and each main concept in each sentence was given a theme, which was then compared to each other in order to determine the main themes and subthemes. Following that, all themes were read carefully several times in order to classify the main themes that had similar meanings in the same category [29]. In the next stage, research objectives and questions were edited and revised if required [30]. The data collection process continued to the end of the research, and the researcher collected and analyzed the data simultaneously [31]. These processes continued until reaching data saturation [27]. Data were collected and analyzed simultaneously and by forward and backward methods in line with the research goal, which was determining the predisposing, reinforcing, and enabling constructs as factors affecting the promotion of COVID-19 prevention behaviors among students [32]. The implemented data were arranged and analyzed systematically in five classification stages; in the first stage, the original text was considered as a meaning unit without manipulation. In the second stage, we summarized the main text and put in condensed meaning units while preserving the meaning. The third stage involved coding with one or two long words. In fact, the code acted as a label that exactly explained the purpose of a certain condensed meaning unit. All featured items were corded by using predetermined codes, and any part that could not be classified with the original plan would be given a new code [21]. The fourth stage included the categorization of codes that were related to each other in terms of content or text. In other words, codes were categorized when they described different aspects, similarities, or differences in the content of the text that belonged to each other. The final stage involved the theme, which explained the main meaning and hidden content in two or more categories. Themes were expressing data at an invisible level [30]. In this method, hidden themes and patterns emerged from the content of the study participants' data and were systematically categorized [31]. Classifications were made based on similarities, and classes were then subdivided into predisposing, reinforcing, and enabling factors [33]. Therefore, the codes were determined over an exploratory process, and predisposing, reinforcing, and enabling factors were identified as the fundamental themes [34]. The research team attempted to establish homogeneity in classes and heterogeneous between the categories [35].

### 2.4. Trustworthiness of Data

In this study, they achieve the credibility that were established, by control, by experts (faculty members) during the research and in-depth prolonged engagement with participants and also checked the results with some of the participants. We utilized the external check to confirm dependability, after analyzing the text, it was provided to the panel of experts, and they confirmed the correctness of the process. The peer checks to achieve the confirmability, results were checked with some of the other people like teachers and students, who did not participate in the research, and they confirmed the fitness of the results as well. And in our study, expression of research limitations and comparison of findings from a participant to another during the research and paying attention to the greater diversity of participants in terms of age, marital status, educational level, and job status contributed to achieve the transferability [18].

## 3. Results

A total of 38 subjects (teachers, parents, and students) were entered into the study, the demographic characteristics of whom are presented in Table 1.

The results were classified into three themes of predisposing, enabling, and reinforcing factors and eight categories of perceived sensitivity, perceived intensity, mental beliefs, behavior outcome evaluation, normative beliefs, motivation to follow, control beliefs, and perceived power (Table 2).

### 3.1. Theme 1: Predisposing Factors

Feeling threatened was the first code extracted from the statements of the participants. According to the subjects, students would not adhere to COVID-19 prevention protocols as long as they did not feel threatened. In addition, this lack of attention to the protocols might be due to the belief that this age group is not at risk of the disease. This category of statements was coded with feeling threatened and was allocated to the class of perceived sensitivity and theme of predisposing factors. The seriousness of the risk, perceiving the intensity of the risk, and understanding the consequences of the disease could force students to adhere to COVID-19 prevention protocols. This category of statements was coded with taking the risk seriously and then allocated to the class of perceived intensity and theme of predisposing factors. Based on the statement of the participants, it became clear that a positive attitude toward COVID-19 prevention behaviors could encourage people to adopt these behaviors. This class of participants' statements was labeled with the code of expectation of the outcome of the behavior. These codes were then added to the category of mental beliefs and allocated to the theme of predisposing factors.

The importance of performing the behavior was another extracted code. According to the subjects' statements, agreeing with the results of performing behaviors that prevent COVID-19 and believing in the desired outcome of performing these behaviors can encourage people to adopt such behaviors. This code was allocated to the class of behavior outcome evaluation and then to the theme of predisposing factors. Based on the viewpoints of the participants, difficulties related to the performance of the behaviors, the disturbances caused by the use of protective equipment, or the ease of not using these tools reflected the causes and barriers to the use of COVID-19 prevention behaviors. These items were coded with the difficulty of performing the behavior and then allocated to the category of perceived power and the theme of predisposing factors since they mostly referred to the person's ability to eliminate obstacles. After receiving the subjects' opinions in this section, it was interesting that when we asked them whether they had suggestions to eliminate these barriers or not, they repeated items classified in categories of perceived sensitivity, perceived intensity, mental beliefs, behavior outcome evaluation, normative beliefs, and motivation.

### 3.2. Theme 2: Enabling Factors

Accompanying of parents, teachers, and classmates, having a monitoring program, and preparing protective equipment were among the issues mentioned by participants that could improve COVID-19 prevention behaviors in students. These items were allocated to the code of behavior facilitation and then to the category of control beliefs and enabling themes.

### 3.3. Theme 3: Reinforcing Factors

Approval and disapproval of behavior by other important people were the code extracted from this part of the participants' statements. Participants believed that there are people whose words and behaviors are important for students. They stated that students would accept and even perform a behavior when they see that other important people such as friends, parents, and teachers accept these behaviors. This code was allocated to the theme of reinforcing factors in the class of normative beliefs. Participants stated that if students believe that important people, such as parents, friends, and teachers, expect them to do something, it will become important to them. Therefore, the importance of the opinions of others was another code that could have a proper effect of adopting COVID-19 prevention behaviors among students. This code was considered as the theme of reinforcing factors after being placed in the class of motivation to follow.

## 4. Discussion

Even though COVID-19 is a communicable disease with a rapid transmission rate, performing prevention behaviors can extremely contribute to its control. However, the key point in doing these behaviors is identifying the components affecting them and laying the foundation for voluntarily adhering to the related protocols. According to the results of the present study, various factors and components affect the promotion of COVID-19 prevention behaviors. In the present study, the mental belief of risk of the virus, which was coded with perceived sensitivity, was one of the factors affecting the promotion of prevention behaviors. However, Wang et al. showed that despite the low perception of risk among participants, they engaged in preventive behaviors [36]. Meanwhile, other studies have shown that prevention behaviors are adhered to when people have a higher risk perception [37, 38]. Another issue detected was students' perception of the seriousness of the risk and consequences of the disease, which were believed to improve prevention behaviors in students. Numerous studies have addressed this issue as an influential component [36, 39]. Therefore, it could be concluded that the perception of the risk could affect the person's acceptance and exploitation of prevention behaviors [40].

Our findings show that students do not feel very risky, and perhaps this finding suggests a link between age and risk perception. Consistent with our findings, Li et al. marked a relationship between the severity of risk perception and age, showing that there was less sensitivity at lower ages [41]. Accordingly, the research team believes that informing students of the amount of risk properly will affect their adherence to prevention behaviors and disease transmission control. In the present study, we extracted two codes of expectation of the behavior outcome and the importance of performing the behavior as attitude toward COVID-19 prevention behaviors from the statements of the participants. According to Mamo et al., understanding the benefits of behaviors played a role in sustainable adherence to performing the behaviors [42]. Other studies have approved this issue, expressing that a positive attitude toward the behavior could be an effective component of adherence to the behavior [43, 44]. Meanwhile, the study of Park and Oh marked that students' attitude toward the behavior had no significant effect on their intention to perform prevention behaviors [45]. This lack of consistency between the results might be due to the difference in research methods.

Normative beliefs and motivation to comply were among the other effective components that the participants emphasized and stated that these will be very influential factors for students to perform protective behaviors against COVID-19 at school. Other similar studies have mentioned the effect of peers and social media on the promotion of COVID-19 prevention behaviors [36, 39]. Moreover, Park and Oh believed that the students intended to imitate the behaviors of other important people in their lives [45]. Parental involvement in the provision of protection equipment was one of the facilitators of the behavior, which was expressed by a large number of participants. According to our findings, providing protective equipment could facilitate the performance of COVID-19 prevention behaviors among the students. The availability of facilities as factors affecting the adoption of behavior was also considered in other study [46]. Based on the views of the participants, feeling discomfort when using protective equipment or having difficulty when applying these tools was among the barriers to the performance of COVID-19 prevention behaviors. This is classified in the perceived power category, and if it is considered an equivalent for self-efficacy, we will see that Costa has regarded it as a strong predictor of behavior change [47], and the greater the barriers, the lower the self-efficacy and possibility of performing the behavior [48]. According to the results obtained by Sim et al., discomforts caused by masks were a barrier to the use of this protective equipment [49]. In order to increase cooperation, it is suggested that alternatives can be designed and also products to increase comfort and reduce problems. In this regard, Abtahi has suggested the use of appropriate alternatives to promote behaviors [50].

## 5. Conclusion

According to the results of the present study, the promotion of COVID-19 prevention behaviors among students was affected by multiple factors. Therefore, designing and implementing effective interventions based on health education theories and models and promotion of health is among the most important issues that determine the promotion of prevention behaviors. Educational and intervention programs that seek success can be designed and implemented based on recognizing the components that affect behavior change. As such, our findings could be used as a guide to design educational interventions aiming at promoting COVID-19 prevention behaviors among students.

## 6. Research Limitations

One of the limitations of the present study was the lack of assessment of other environmental components such as organizational and political factors. Another limitation was the financial situation of families, which was not mentioned due to ethical issues. The epidemic conditions of COVID-19 and the existing restrictions for preventing the transmission of the disease led to limitations in the presence of people and participate in interviews, such that about six individuals refused to be interviewed and the research team was forced to choose other people. Even though perceived power was considered as a component, it was better that self-efficacy was also coded and analyzed as a separate component. Another limitation was the probability of dishonesty of some of the participants in answering the questions of interviews.

## 7. Ethical Considerations

The research project was approved by the ethics committee of Shiraz University of Medical Sciences with the code of ethics of IR.SUMS.REC.1400.135. Participation in the study was voluntary, and the subjects were entered into the study following receiving informed consent from them. Interviews, note taking, and recording the voice of the subjects would not happen in case of lack of desire of the participants. In addition, they were ensured of the confidentiality terms regarding their personal information, meaning that the collected data was presented anonymously.

## Figures and Tables

**(a):** 

Demographic characteristics of teachers and parents							
Age	*N*	Gender	*N*	Occupation	*N*	Level of education	*N*
25-34 years	10	Male	10	Teacher	5	PhD	1
35-44 year		Female	15	Principal	4	MSc	6
45-54 years	9			Health instructor	6	BSc	13
	6			Parents	10	High school	5

**(b):** 

Demographic characteristics of students			
Academic grade	*N*	Gender	*N*
Seventh grade	4	Male	13
Eighth grade	5		
Ninth grade	4		

**Table 2:** Study findings based on participants' perspectives.

Participant	Meaning unit	Condensed meaning units	Code	Category	Theme
38-year-old mother	Maybe it is because some students overlook the disease.	Students overlooked the importance of the disease.	Feeling threatened	Perceived sensitivity	Predisposing factors Enabling factors
44-year-old father	Students do not properly think about the risks of the disease and what problems it creates for the family.	Students do not think about the risks of the disease			
39-year-old father	One of the best things to do is to tell the children the truth and give the student an example to follow. For example, we could say that someone has been diagnosed with the disease and is ill or dead to adhere to the protocols.	Tell the children that someone has been diagnosed with corona disease and is ill or dead so that they adhere to the protocols.	Taking the risk seriously	Perceived intensity	
Eighth grade student	I am afraid to go to school because I fear that I will get the disease and be hospitalized. If I go, I will wear a mask and observe my distance.	I am afraid that the person will be hospitalized; so, I will adhere to the protocols.			
29-year-old health instructor	As a health educator, I need to make it clear to children that following protocols can help prevent the disease.	Adherence to protocols can prevent the disease.	The expectation of the outcome of the behavior	Mental beliefs	
Ninth grade student	My suggestion is that following the protocols is very important and can help a lot not to get sick. I am sure if we follow it, I will not get sick.	Adherence to protocols helps not to get sick.			
51-year-old father	Of course, if the health of the family members is important for the student, they must observe the protocols.	If family health is important to the student, they will adhere to the protocols.	Importance of performing the behavior	Behavior outcome evaluation	
39-year-old teacher	At school, we should encourage students to take interest in the health of themselves, their family members, and even their classmates. In a way, we must teach them to be responsible.	The health of themselves and their family members and even their classmates is important to them.			
Eighth grade student	I will not do it because it is not comfortable. Wearing a mask from morning to noon is very annoying. Is it easier not to wear it because it hurts the behind of my ears.	Wearing a mask from morning to noon is very annoying. It is easier not to wear it.	The difficulty of performing the behavior	Perceived power	
Seventh grade student	Disinfectants have a bad odor and damage the skin. When something makes a person's skin rough, it becomes difficult to use.	Disinfectants smell bad and damage the skin.			
51-year-old teacher	An important issue for parents is to help in providing equipment, masks, gloves, and disinfectants.	Supporting parents in providing protective equipment	Facilitation of the behavior	Control beliefs	
42-year-old mother	Planning is the most important thing that can lead children to adhere to protocols. For example, a supervisory and control plan can be developed by teachers.	Supervisory and control planning by teachers			Reinforcing factors
Ninth grade student	Some of the students mock us when we adhere to the protocols. They bully us and call us cowards. Therefore, we no longer wear masks.	People mock me when I adhere to the protocols.	Behavior confirmation	Normative beliefs	
	The cooperation of parents is more important than anything. They should take the issue seriously, observe it themselves, and tell the children that the danger is near, and we must follow health protocols to get rid of this danger.	Parents should take the issue seriously and tell their children to comply.			
40-year-old mother	The student has a special respect for the teacher. If the teacher asks them, I am sure that they will wear a mask, wash their hands, and even observe the distance.	The student respects the teacher and will comply if asked by the teacher.	The importance of the opinion of others	Motivation to follow	
36-year-old teacher	Children will adhere to the protocols if their parents do as well. Most of the time, children wait to see how their parents deal with a situation. For instance, if a father wears a mask, the child will realize that it is important to do so.	Their father wears a mask, and they realize that it is important to do so.			

## Data Availability

The data used to support the findings of this study are included in the paper. However, if additional data is required, they are available from the corresponding author upon request.
